# Pretrained Transformer Language Models Versus Pretrained Word Embeddings for the Detection of Accurate Health Information on Arabic Social Media: Comparative Study

**DOI:** 10.2196/34834

**Published:** 2022-06-29

**Authors:** Yahya Albalawi, Nikola S Nikolov, Jim Buckley

**Affiliations:** 1 Department of Computer Science and Information Systems University of Limerick Limerick Ireland; 2 Department of Computer and Information Sciences College of Arts and Science University of Taibah Al-Ula Saudi Arabia; 3 The Irish Software Research Centre, Lero University of Limerick Limerick Ireland

**Keywords:** social media, machine learning, pretrained language models, bidirectional encoder representations from transformers, BERT, deep learning, health information, infodemiology, tweets, language model, health informatics, misinformation

## Abstract

**Background:**

In recent years, social media has become a major channel for health-related information in Saudi Arabia. Prior health informatics studies have suggested that a large proportion of health-related posts on social media are inaccurate. Given the subject matter and the scale of dissemination of such information, it is important to be able to automatically discriminate between accurate and inaccurate health-related posts in Arabic.

**Objective:**

The first aim of this study is to generate a data set of generic health-related tweets in Arabic, labeled as either accurate or inaccurate health information. The second aim is to leverage this data set to train a state-of-the-art deep learning model for detecting the accuracy of health-related tweets in Arabic. In particular, this study aims to train and compare the performance of multiple deep learning models that use pretrained word embeddings and transformer language models.

**Methods:**

We used 900 health-related tweets from a previously published data set extracted between July 15, 2019, and August 31, 2019. Furthermore, we applied a pretrained model to extract an additional 900 health-related tweets from a second data set collected specifically for this study between March 1, 2019, and April 15, 2019. The 1800 tweets were labeled by 2 physicians as *accurate*, *inaccurate*, or *unsure*. The physicians agreed on 43.3% (779/1800) of tweets, which were thus labeled as *accurate* or *inaccurate*. A total of 9 variations of the pretrained transformer language models were then trained and validated on 79.9% (623/779 tweets) of the data set and tested on 20% (156/779 tweets) of the data set. For comparison, we also trained a bidirectional long short-term memory model with 7 different pretrained word embeddings as the input layer on the same data set. The models were compared in terms of their accuracy, precision, recall, F_1_ score, and macroaverage of the F_1_ score.

**Results:**

We constructed a data set of labeled tweets, 38% (296/779) of which were labeled as inaccurate health information, and 62% (483/779) of which were labeled as accurate health information. We suggest that this was highly efficacious as we did not include any tweets in which the physician annotators were unsure or in disagreement. Among the investigated deep learning models, the Transformer-based Model for Arabic Language Understanding version 0.2 (AraBERTv0.2)-large model was the most accurate, with an F_1_ score of 87%, followed by AraBERT version 2–large and AraBERTv0.2-base.

**Conclusions:**

Our results indicate that the pretrained language model AraBERTv0.2 is the best model for classifying tweets as carrying either inaccurate or accurate health information. Future studies should consider applying ensemble learning to combine the best models as it may produce better results.

## Introduction

### Background

In the past 2 decades, there has been a dramatic increase in the number of people who use social media (SM) to participate in discussions on various topics, such as politics [1], health [2], and education [3]. Regarding health-related information, several recent studies from Saudi Arabia found that Twitter is the preferred SM platform for communicating and accessing medical information. For example, it was preferred by orthopedic surgeons to reply to (personal and professional) medical questions [4], by dental practitioners for medical consultations [5], by patients with diabetes to search for health information [6], by female students at a university in Saudi Arabia to read about systemic *lupus erythematosus* [7], and by adolescents to search for oral health information [2].

A significant problem with this form of communication is that there is no quality control over the medium, and most of the health information presented on Twitter seems inaccurate, as illustrated by the various studies summarized in Table 1. Indeed, multiple data science studies have used data sets of health-related communication on SM to study this phenomenon, and some studies [8-10] went further to design frameworks for detecting the accuracy of health information on SM.

**Table 1:** Summary of studies that analyzed the accuracy of health information on social media.

Studies	Number of tweets or documents	Sources	Methods to label	Language covered	Percentage of the accuracy	Topics covered	Type of study
Swetland et al [11]	358	Twitter	Expert votes; relabeling in cases of disagreement	English	25.4% inaccurate	COVID-19	Exploratory
Albalawi et al [12]	109	Twitter	Two physicians; delete if there is a disagreement	Arabic	31% inaccurate	General	Quantitative pilot study
Saeed et al [8]	208	Twitter	Expert votes; relabeling in cases of disagreement	Arabic	38% inaccurate	Cancer	ML^a^
Sharma et al [13]	183	Facebook	Two physicians; delete if there is a disagreement	English	12% inaccurate	Zika	Quantitative
Alnemer et al [14]	625	Twitter	Vote if the experts do not agree	Arabic	50% inaccurate	Only tweets from health professionals	Quantitative and exploratory study
Zhao et al [10]	5000	Health forum	Annotator voting; in addition, consulted an expert to validate information labeled as misleading	Chinese	11.4% misinformation	Autism	ML
Sell et al [15]	2460	Twitter	Coders checked the interagreement on 200 tweets	English	10% inaccurate	Ebola	Quantitative
Chew and Eysenbach [16]	5395	Twitter	Coder checked agreement on 125 tweets; unsubstantiated by the following reference standards: the CDC^b^ and Public Health Agency of Canada for scientific claims and a panel of credible web-based news sources (eg, CNN^c^ and BBC^d^) for news-related claims	English	4.5% inaccurate	H1N1	Exploratory
Sicilia et al [9]	800	Twitter	Annotator’s agreement; relabeling in cases of disagreement; here, the definition for misinformation was “news items without a source”	English	Unknown	Zika	ML
Kalyanam et al [17]	47 million	Twitter	Type of hashtags	English	25% of the analyzed tweets were speculative	Ebola	Quantitative
Al-Rakhami and Al-Amri [18]	409,484	Twitter; keywords	Although they used coders, their definition of a rumor included lack of a source; hence, unconfirmed information was automatically classified as uncredible; in addition, tweets were classified by only 1 coder who checked interagreement on 20 tweets	Not noted, but the keywords were in English	70% uncredible	COVID-19	ML
Elhadad et al [19]	7486	Various websites	Fact-checking websites and official websites	English	21%	COVID-19	ML
Seltzer et al [20]	500	Instagram	Coders’ agreement	English	23%	Zika	Exploratory
Ghenai et al [21]	26,728	Twitter	Defined keywords to the extracted tweets based on rumors identified from the WHO^e^ website; then, the coders labeled the tweets	English	32%	Zika	ML

^a^ML: machine learning.

^b^CDC: Centers for Disease Control and Prevention.

^c^CNN: Cable News Network.

^d^BBC: British Broadcasting Corporation.

^e^WHO: World Health Organization.

Previous studies have focused on specific health issues and sometimes on specific types of rumors [8,18,19,21,22]. This suggests the need for a more general framework that can detect the accuracy of health information across known and previously unknown health conditions, such as during the outbreak of a previously unknown infectious disease.

Given the prevalent use of Twitter for the spreading of health information in Saudi Arabia [2,4-7,23,24], we aimed to inform the development of a new and more generic framework that is not bound to a specific disease or rumor type and detect the accuracy of a broad base of health-related tweets in Arabic.

### Related Work

In this section, we review the methods used to label health-related tweets as either accurate or inaccurate to create labeled data sets. We also review previously proposed machine learning (ML) models for detecting the accuracy of health-related tweets, including deep learning (DL).

#### Methods Used to Label Health-Related Tweets

Studies addressing the accuracy of health-related tweets can be classified into 3 groups. The first group comprised studies that labeled health-related tweets according to the information they contained, regardless of the source of the information. The second group comprised studies that relied on external (fact-checking or very reputable) websites. The last group comprised studies that relied on various characteristics of the tweets or only on the source of the information to judge the accuracy of the tweets.

Regarding the concepts of accuracy and misinformation, Chou et al [25] defined *misinformation* as information that lacks scientific evidence. A more precise definition can be found in the study by Tan et al [26], where the authors defined inaccurate information or misinformation as “explicitly false,” according to what would be deemed incorrect by expert consensus. In the study by Nyhan and Reifler [27], the authors combined these definitions to describe misinformation or inaccurate health information as information that is not supported by clear evidence and expert opinion.

Studies relying on the opinions of experts seemed to indirectly or directly use these definitions to assess accuracy; however, it should be noted that, although misinformation is inaccurate, it is not necessarily intended to be so. In contrast, disinformation is information that is intentionally deceptive [28]. Examples of *opinions of experts* studies are included in Table 1 [8,10,11,14]. These involved labeling health-related tweets based on the opinions of health experts. The tweets were labeled as inaccurate or accurate by at least two experts. A third expert was typically involved when there was a disagreement between the original 2 experts: this expert cast the deciding vote for controversial tweets.

Vraga and Bode [29] criticized the abovementioned definition of misinformation, raising the point that there are many issues on which experts do not agree. However, they state that as long as there is more evidence supporting the information, the agreement rate between experts will increase. Taking a stricter approach, Albalawi et al [12] and Sharma et al [13] excluded tweets on which experts disagreed in an attempt to exclude uncertainty from their data sets. Table 1 summarizes these studies.

Unsurprisingly, studies that relied on expert opinion used relatively small data sets (ranging from 109 to 625 tweets) compared with studies that used other labeling methods (Table 1). Even those that used nonexperts but used manual coding (performed by nonexpert annotators) tended to work on a small sample of the data set [9,20].

The second group comprised studies that relied on an external website, such as a fact-checking website, to label the tweets. One such example is the study by Elhaddad et al [19], which relied on a fact-checking website to identify misleading information. A similar method was used by Ghenai et al [21], who relied on the website of the World Health Organization (WHO) to identify 6 rumors. From these rumors, they derived keywords to extract relevant tweets. The drawback of this method is that only tweets relevant to specific rumors were extracted; thus, the model was trained only on this limited number of rumors. Furthermore, these methods are highly language restricted: both studies referred to in Table 1 were performed in English, as mandated by the WHO website and the fact-checking website.

Other methods relied on various characteristics of the tweets or only on the source of the information without judging the actual information. For example, in the study by Kalyanam et al [17], the authors identified tweets as credible if they included hashtags that indicated that they originated from noted agencies or other reliable sources, and tweets were identified as speculative if they included hashtags that implied an increase in fear, rumors, or scams.

Similarly, Sicilia et al [9], Al-Rakhami and Al-Amri [18], and Chew and Eysenbach [16] defined credible tweets as tweets that have information from a confirmed, reliable source, such as the WHO, Centers for Disease Control, or another official health agency. This method differs from the method used by the second group mentioned previously as it first identified a tweet and then examined its source. In contrast, the methods in the second group first identified a trustworthy website and then used the information on the website to identify tweets of interest.

More generally, Yin et al [30] stated that a website is *trustworthy* if it provides correct information and suggests that information is likely to be true if it is provided by a trustworthy website. Studies that relied on trustworthy websites to identify rumors [9,18,21] seemed to follow this definition, even if they did not explicitly state it.

It should be noted that based on the data in Table 1, all Arabic studies that relied only on expert opinion [8,12,14] were small scale and qualitative; therefore, it would be impossible to scale them up. Notably, the percentage of inaccurate tweets for English studies that rely on expert opinions is in the range of 10% to 25%, whereas the corresponding range for Arabic studies is 31% to 50%. This finding suggests a greater occurrence of inaccurate health-related tweets in Arabic than in English.

#### ML Approaches

Of the 14 studies reported in Table 1, which analyzed the accuracy of health-related tweets in general, 6 (43%) proceeded to train an ML model to detect the accuracy of health information, as shown in Table 2.

**Table 2:** Summary of studies that developed MLa models to detect the accuracy of health-related information.

Study	ML approach	Results	Labeling type
Elhadad et al [19]	Deep learning multimodel, GRU^b^, LSTM^c^, and CNN^d^	99.99% (F_1_ score)	Ground truth data from websites
Ghenai et al [21]	Random forest	94.5% (weighted average for F_1_ score)	Crowdsource agreement but keywords are based on 4 WHO^e^ website-identified rumors
Al-Rakhami and Al-Amri [18]	Ensemble learning and random forest+SVM^f^	97.8% (accuracy)	Single annotator only after confirming source
Zhao et al [10]	Random forest	84.4% (F_1_ score)	Annotator vote; in addition, consulted an expert to validate misleading information
Sicilia et al [9]	Random forest	69.9% (F_1_ score)	Agreement of a health expert
Saeed et al [8]	Random forest	83.5% (accuracy)	Agreement of a health expert

^a^ML: machine learning.

^b^GRU: gated recurrent unit.

^c^LSTM: long short-term memory.

^d^CNN: convolutional neural network.

^e^WHO: World Health Organization.

^f^SVM: support vector machine.

Studies reporting on training ML models included Elhadad et al [19] and Al-Rakhami and Al-Amri [18], who used ensemble learning on an English data set. Elhadad et al [19] used ensemble learning that involved multiple DL architectures, and Al-Rakhami and Al-Amri [18] trained ensemble models comprising traditional ML algorithms, such as support vector machine (SVM) and random forest (RF). Another similarity between these studies is the method used to identify misleading information. Elhadad et al [19] built their data set by extracting ground truth data and rumors from fact-checking websites. Al-Rakhami and Al-Amri [18] considered tweets credible if they have a reliable source and misleading otherwise. Both models reported a high level of accuracy (>97%), as shown in Table 2.

From Tables 1 and 2, it is clear that studies that relied on a fact-checking website [19,21] and studies that determined the accuracy of a tweet based on its source [18] obtained a high level of accuracy, possibly as these models were trained on relatively large data sets.

For example, Al-Rakhami and Al-Amri [18] trained their model using 409,484 tweets. However, automated labeling left open the possibility of incorrect labeling, and all these studies were conducted in English.

Most of the studies that developed ML models focused on outbreaks (4/6, 67% of studies). Studies that developed ML models for nonoutbreak conditions [8,10] obtained less accurate results compared with outbreak conditions. This might be because these nonoutbreak condition models were trained on a limited number of documents compared with the outbreak models. We also found that the level of accuracy obtained for nonoutbreak data sets was approximately 84% (Table 2). It is also notable that all of these studies trained an RF model.

Table 2 (and our associated literature review) suggests that recent advancements in DL have not been sufficiently applied to the detection of misleading Arabic health information. In our previous work, we have shown that DL architectures using word embedding as an input layer outperform other traditional ML models, such as SVM and naive Bayes, in the detection of Arabic health-related information on SM [31]; however, in this paper, we move past that to the classification of Arabic health-related tweets based on their accuracy.

Word embedding is a learned representation of words in natural language processing (NLP) [32]. Words with similar meanings typically have similar numbers in their vectors. The closer the words are in meaning, the shorter the distance between the 2 vectors representing them. One of the main criticisms of the word embedding approach is that it is considered context free; that is, the embedding of a word is not affected by its position in the sentence [33]. Hence, it is also referred to as static word embedding. However, in practice, the meaning of a word may depend on its position in a sentence.

In recent years, pretrained language models have been proven to work well for many NLP tasks, including entity recognition, language translation, and text classification [34]. Unlike static word embedding techniques, such as Skip-Gram and Continuous Bag of Words, language models can learn the context of the words and thus assign different values for the words depending on their context [33]. There are different types of language models, including contextual word vectors and embeddings from language models [33]. One of the most popular language models is the bidirectional encoder representations from transformers (BERT), which has been proven to perform well in text classification tasks.

The superiority of transformer models compared with other text classification methods is well documented, especially in the recent literature. Multiple studies have compared transformer models with other DL models [35-39], and the results showed that transformers outperformed the ML models, including different DL architectures and traditional ML models, such as SVMs and RF. This indicates the potential capability of transformers to better detect the accuracy of Arabic health information on SM.

Therefore, in this study, we aimed to contribute to this field by developing a data set of certified accurate or inaccurate Arabic health-related tweets and investigating the ability of the BERT or pretrained word embedding model to detect the accuracy of Arabic health-related tweets across a wide range of health-related issues.

## Methods

### Overview

The empirical method comprised 2 parts. The first part addressed the extraction of health-related tweets using the model proposed in our previous study [31]. In that study, we used a health lexicon that focused more on general health keywords rather than specific outbreaks, as a recent study suggested that general health misinformation is more likely to spread than, for example, COVID-19 [40]. In contrast, Table 1 illustrates that most studies in this area focused on a specific domain or disease outbreak.

**Figure 1:**
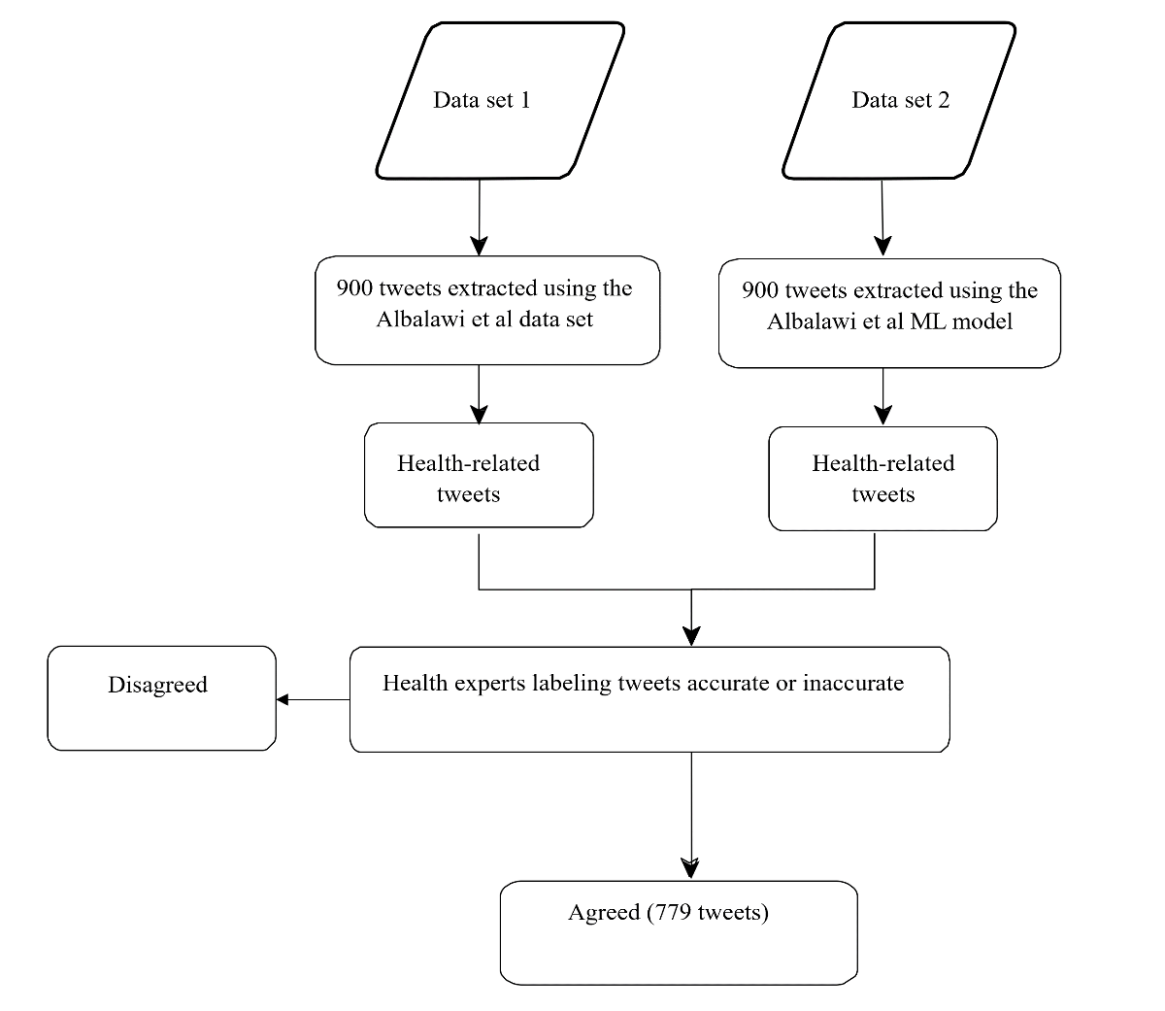
Overview of the process followed in labeling tweets as either accurate or inaccurate [31]. ML: machine learning.

The extracted health-related tweets were labeled by health experts as either accurate or inaccurate. Figure 1 presents an overview of this portion of the study.

In the second part, we propose 2 types of trustworthiness—detecting models to automatically classify health-related tweets as either accurate or inaccurate—and evaluate them: bidirectional long short-term memory (BLSTM) DL models and pretrained transformer language models.

### Building Data Sets of Trustworthy Health-Related Tweets

In this study, we used 2 data sets containing health-related tweets. The first data set was the result of our previous study [31].

The first data set was extracted from 297,928 tweets posted between July 15 and August 31, 2019. Of these 297,928 tweets, 5000 (1.68%) were randomly sampled and labeled by 2 annotators as either *health-related* or *not health-related*. A third annotator resolved disagreements between the 2 annotators.

The first data set was extracted during the summer holidays in Saudi Arabia for 45 consecutive days. To assess generality, we extracted the second data set for a different timeframe: during *Hajj* and *Eid al Adha* (Muslim holy days) and during school days between March 1 and April 15, 2019. The second set of 900 tweets used the ML methodology proposed in the same study [31], as the availability of health professionals was constrained by the ongoing COVID-19 pandemic and the ML model derived in that study achieved a high-quality result (93% accuracy).

The methodology proposed in the study by Albalawi et al [31] comprised extracting tweets from a set of collected tweets with the help of a health lexicon and then further filtering out tweets not related to health with the help of an ML model. On the basis of the health lexicon, 217,702 tweets were extracted. Of the 217,702 tweets, we sampled 5000 (2.3%) tweets and applied the ML model to extract 900 (0.41%) health-related tweets.

Finally, we added 900 tweets from the second data set to 900 tweets sampled from the first data set and had those 1800 tweets labeled as either accurate or inaccurate health information by 2 medical physicians.

### Labeling Accurate or Inaccurate Tweets

The physicians were asked to manually label each of the 1800 health-related tweets into one of the following categories: *accurate health information*, *inaccurate health information*, and *not sure about the accuracy*.

We followed the protocol of relying on the opinions of experts to define the accuracy of the information collected. Taking into account the points made by Vraga and Bode [29], every tweet was assessed by 2 experts, and a tweet was included in the final data set for this study only if both experts agreed on its accuracy; that is, we reduced uncertainty by excluding information that was not sanctioned by all experts (indeed, later show that between-physician reliability in this coding was limited, buttressing the need for increased certainty when using human classification, as stated by Vraga and Bode [32]). The *not sure* option was offered to the physicians to avoid forcing them to evaluate the tweets if they did not have enough relevant health knowledge to accurately evaluate them or if the tweets were ambiguous.

Although other studies invited a third annotator to resolve disagreements, our approach was stricter in reducing uncertainty in the data set by excluding tweets for which there was a disagreement between the 2 annotators. Of 1800 tweets, the 2 physicians agreed on 779 (43.3%) tweets, which were labeled as containing either accurate or inaccurate health information. The physicians disagreed on 9.1% (163/1800) of tweets. The remaining 47.7% (858/1800) of tweets were labeled as *unsure* by at least one physician. We dropped the tweets on which at least one of the physicians was unsure and used the remaining 779 tweets in our experiments.

Although the 779 tweets constituted a relatively small data set, most of the data sets constructed in the literature based on agreements between health experts were relatively small. As shown in Table 1, the highest number of health-related tweets judged by health experts in other studies was 625 in the study by Alnemer et al [14].

These 779 tweets, labeled as either accurate or inaccurate, can be found in Multimedia Appendix 1. Please note that we only share tweet IDs and labels as the Twitter policy prevents the content of the tweets from being redistributed. These tweet IDs can be used to obtain the text of tweets using the Twitter application programming interface [41].

### Considered DL Models

#### Overview

**Figure 2:**
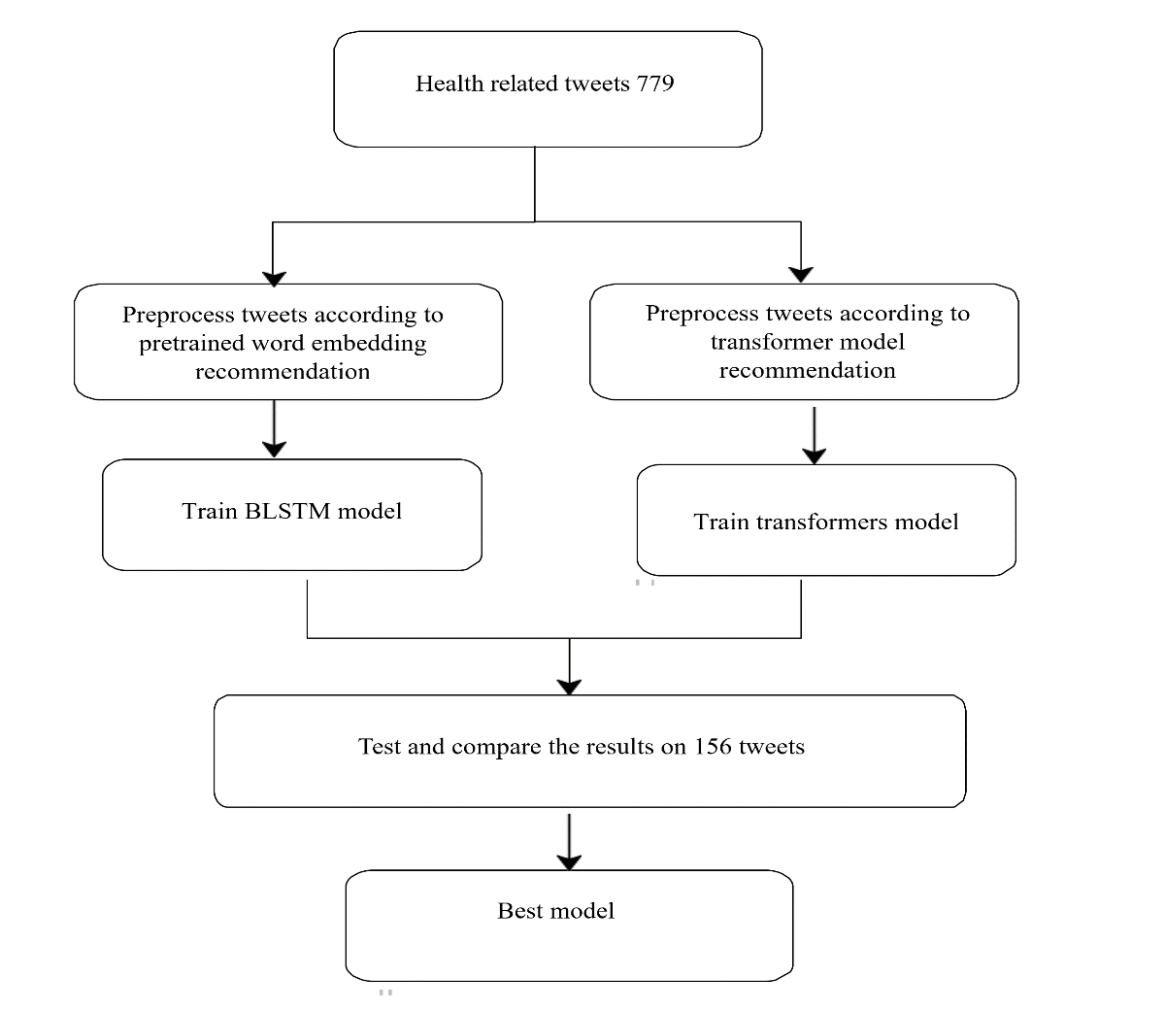
Overview of the process used to train and select machine learning models. BLSTM: bidirectional long short-term memory.

After completing the annotation of the health-related tweets as either accurate or inaccurate, we trained 16 classification models, 7 (44%) of which used a BLSTM architecture with pretrained word embeddings as their input layers, and 9 (56%) of which used a pretrained transformer language model. Figure 2 illustrates the steps implemented during this stage. Further details are provided in the following sections.

#### The BLSTM Architecture

For 44% (7/16) of the trained models, we used a BLSTM architecture with pretrained word embeddings as the input layer. Long short-term memory (LSTM) is a type of recurrent neural network that takes advantage of dependencies between parts of the input sequence and can learn these dependencies. LSTM also preserves the information of past input. The BLSTM variation differs from LSTM because of its ability to learn the dependencies between past and future elements [42]. BLSTM has been found to perform well in many NLP tasks, including text classification [43]. The BLSTM model begins with input and embedding layers to which a dropout layer is added, followed by a BLSTM layer with another added dropout layer [31]. BLSTM has been shown to perform better than traditional ML models (SVM, naive Bayes, k-nearest neighbors, and logistic regression) and conventional neural networks in a previous study on detecting Arabic health-related tweets [31]. For the input layer, we used 7 pretrained word embedding models for Arabic [44-47]. It should be noted that AraVec, Mazajak, and ArWordVec come in 2 variations: Continuous Bag of Words and Skip-Gram and, finally, BLSTM fastText.

#### Transformer Models

BERT is a transformer language model that has shown superiority in many NLP tasks.

Different Arabic pretrained language models exist, which are based on transformers that have been developed recently by the Arabic NLP community. Most of these pretrained language models were built on top of the BERT-base model. Some of them also provided a version based on BERT-large.

The difference between BERT-base and BERT-large is that BERT-base uses 12 layers, 768 hidden layers, 12 heads, and approximately 136 million parameters, whereas the BERT-large model uses 24 layers, 1024 hidden layers, 16 heads, and approximately 370 million parameters [48]. All models may not leverage BERT-large as it is more difficult to train and comes with a higher computational cost than BERT-base [49].

Examples of pretrained Arabic language representation models that offer both base and large variants are ArabicBERT [50] and Transformer-based Model for Arabic Language Understanding (AraBERT) [51]. AraBERT was considered the first Arabic-specific transformer language model introduced in 2020 by Antoun et al [51]. In 2021, an updated version of AraBERT was released [52]. AraBERT is considered one of the best transformer language models for NLP, outperforming other models for Arabic sentiment analysis [53]. AraBERT version 2 (AraBERTv2) preprocesses text using Farasa segmentation. Farasa segmentation involves breaking the words based on the prefix and suffix [54], whereas AraBERT version 0.2 (AraBERTv0.2) preprocesses the text without using Farasa segmentation. In this study, we experimented with these 6 models: AraBERTv2, AraBERTv0.2, and ArabicBERT in both variants of BERT (base and large).

In addition to 6 models, we also investigated 3 other state-of-the-art pretrained language models, namely QARiB [55], MARBERT, and ARBERT [56], which are based only on BERT-base. These models reportedly perform well on text classification tasks [50,55-57]. Table 3 summarizes the characteristics of the pretrained language models used in this study.

**Table 3:** Pretrained language models.

Name	Basis	Size	Corpus
ARBERT [56]	BERT^a^-base	61 GB of MSA^b^ text (6.5 billion tokens)	Books and news (news and Wikipedia articles)
MARBERT [56]	BERT-base	128 GB of text (15.6 billion tokens)	1 billion Arabic tweets
QARiB [55]	BERT-base	14 billion tokens; vocabulary: 64,000	420 million tweets and approximately 180 million sentences of text from Arabic Giga Word, Abulkhair Arabic Corpus, and OPUSc
ArabicBERT [50]	BERT-base and BERT-large	95 GB of text and 8.2 billion words	Arabic OSCARd version, Wikipedia, and other resources
AraBERTv0.2^e^ [52]	BERT-base and BERT-large	77 GB, 200,095,961 lines, 8,655,948,860 words, or 82,232,988,358 characters	OSCAR unshuffled and filtered Arabic Wikipedia articles The 1.5 billion words Arabic Corpus The OSIANf corpus Assafir news articles
AraBERTv2^g^ [52]	BERT-base and BERT-large	77 GB, 200,095,961 lines, 8,655,948,860 words, or 82,232,988,358 characters	OSCAR, unshuffled and filtered Arabic Wikipedia articles The 1.5 billion words Arabic corpus The OSIAN corpus Assafir news articles

^a^BERT: bidirectional encoder representations from transformers.

^b^MSA: Modern Standard Arabic.

^c^OPUS: open parallel corpus.

^d^OSCAR: Open Superlarge Crawled Aggregated corpus.

^e^AraBERTv0.2: Transformer-based Model for Arabic Language Understanding version 0.2version 0.2.

^f^OSIAN: Open Source International Arabic News.

^g^AraBERTv2: Transformer-based Model for Arabic Language Understanding version 0.2 version 2.

#### Evaluation Metrics

The F_1_ score, recall, precision, accuracy, and macroaverage of the F_1_ score were used to evaluate the ML models, as detailed in Textbox 1. The macroaveraged F_1_ score is the averaged F_1_ score across all classes, which are accurate and inaccurate health-related tweets [58].

**Metrics used to evaluate the machine learning models.**

**Recall**

- True positives / (true positives + false negatives)

**Precision**

- True positives / (true positives + false positives)

**F_1_ score**

- (2 × precision × recall) / (precision + recall)

**Accuracy**

- (true positives + true negatives) / total sample

**Macroaveraged F_1_ score**

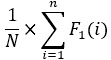
**(1)**, where N is the number of classes

#### Preprocessing Data

In this study, text was preprocessed following the procedure outlined by the authors of the corresponding pretrained word embedding models. Li et al [59] found that this is the best text preprocessing practice when working with pretrained word embeddings. Similarly, for all pretrained word embedding models [44-47] and pretrained language models [50,52,55,56], we followed the steps provided by the original studies.

Of the 779 tweets, we split the data set into training, validation, and test data sets in ratios of 507 tweets (65.1%) for training, 116 tweets (14.9%) for validating the model, and 156 tweets (20%) for testing.

### Ethics Approval

This study did not require institutional review board approval from the Science and Engineering Research committee at the University of Limerick because ethical approval is not required for publicly available data. It should be emphasized, during the study, that any associated text that can be used to identify the authors of the tweets has been removed from the text (eg, @name, user ID).

## Results

### Data Set Description

The κ coefficient for all categories was 0.377, which is in fair agreement according to Cohen [60]. However, the benchmark scale proposed by Fleiss et al [61] to evaluate the agreement indicates that such a coefficient is poor (<0.40=poor, 0.40-0.75=intermediate to good, and >0.75=excellent). Given the low κ coefficients across the 3 categories, we considered only cases where both physicians were explicitly in agreement, as they were on 779 tweets from the original data sets.

Of the 1021 tweets that were excluded, 874 (48.6%) were labeled *not sure* by at least one physician, and in the case of 147 (14.4%) tweets, the physicians disagreed regarding the accuracy of the tweets.

Of the 779 tweets physicians agreed on in our data set, 296 (38%) were labeled as inaccurate and 483 (62%) were labeled as accurate. This finding is similar to the inaccuracies reported in other studies (Table 1).

Textbox 2 presents examples of accurate and inaccurate health-related tweets. As can be seen from the tweets in the textbox, they cover a wide range of topics, including but not limited to psychology and cancer. Interestingly, in the third accurate tweet example, the difficulty for nonexperts in discerning accurate from inaccurate health information is illustrated, as advice against taking antidiarrhea drugs in the event of food poisoning is slightly counterintuitive.

**Examples of inaccurate and accurate health-related tweets.**

**Accurate**

- “Tomorrow enjoys the feast. and get closer to God with your sacrifice
  And eat but do not extravagant and feed the contented and be merciful as God has commanded you
  Eating too much red meat might:
  Raise the level of triglycerides
  Raise cholesterol
  Increase uric salt in the blood Increases gout attacks in the joints”

- “Symptoms of social phobia
  Sometimes, social phobia can be accompanied by physical signs and symptoms, which may include:
  Flashness
  Rapid heart palpitations
  Shivering and sweating
  Upset stomach or nausea
  Difficulty catching breath
  Dizziness or lightheadedness
  Feeling like your mind has gone blank
  Muscle tension”

- “In the event of food poisoning, please take care not to use antidiarrheal medicines, as they may worsen the condition”
- “Hemoglobin is a group of proteins in red blood cells whose function is to transport oxygen from the lungs to the body, return carbon dioxide from the body, and transport it to the lungs and get rid of it through breathing.

Iron is an important element and enters the composition of hemoglobin, so if iron deficiency, hemoglobin decreases, and anemia occurs.”

- “Among the ways to prevent lung cancer:
  Stay away from smoking
  Avoid passive smoking
  Avoid carcinogenic and radioactive materials.”

**Inaccurate**

- “Scientific research,
  The research says that Zamzam water bears the name (water), but it differs radically from water compounds, as all the waters of the world belong to the acidic compound, except for (Zamzam water).
  It is (alkaline!) Glory be to God. There is no other alkaline water on the face of the earth. So, when you drink it in abundance, the human body has a strong immunity against viruses!!”

- “When Western scholars searched for the causes of mental illness, they found only two reasons (fear and sadness) fear of the future and sadness of the past, both of which are the opposite of happiness.”
- “Did you know that a 5-minute tantrum is so stressful that it weakens the immune system for more than 6 hours”
- “Cupping helps smokers to quit smoking or reduce the negative impact on the body through:
  Removing excess hemoglobin from the body by excreting aging red blood cells, and thus the disappearance of the pathological symptoms of high hemoglobin caused by smoking”

- “Just a spoonful of cinnamon daily:
  Rich in anti-inflammatory and antioxidants
  Prevents all types of cancer
  Prevents heart disease
  Anti-diabetes”

Some tweets claimed the benefits of some traditional foods and spices. For example, some tweets promoted *Zamzam* (holy water for Muslims), claiming there was scientific research that stated that it could strengthen the human immune system; experts classified the information as inaccurate.

In addition, the examples of accurate tweets presented here suggest that accurate health-related tweets tend to be more preventive in nature, a finding supported by the wider sampling of accurate tweets. As shown in Textbox 2, the accurate tweets advised users to stop eating too much red meat as it causes gout or increases cholesterol, stop smoking to prevent lung cancer, and stop taking anti-inflammatory drugs in the event of food poisoning. In contrast, as noted earlier, inaccurate tweets promoted natural and alternative medicine such as curbing eating and drinking *Zamzam* water for their health benefits. An interesting example was in relation to cancer, where accurate tweets advised readers to stop smoking; however, some of the inaccurate tweets were also preventive, and they advised taking a spoonful of cinnamon to prevent all types of cancer.

### DL Models

In terms of the comparison of models, we observed that overall, BERT models performed better than BLSTM models based on the accuracy and the F_1_ score for both classes (when referring to the metric accuracy in this section, we will call it *model accuracy* to disambiguate it from the accurate or inaccurate classification). Overall, AraBERTv0.2-large performed better than all other models. Specifically, the best model was AraBERTv0.2-large (macro F_1_ score 87%), followed by AraBERTv2-large (macro F_1_ score 86%) and AraBERTv0.2-base (macro F_1_ score 85%), as shown in Table 4. These findings hide larger but still small variations in the precision and recall scores of individual techniques for inaccurate and accurate tweets. For example, although AraBERTv0.2-base achieved a recall of 78% for inaccurate tweets, AraBERTv0.2-large achieved a recall of >83%.

The results also suggest that, in general, BERT-large models tended to be better at detecting inaccurate tweets than the BERT-base models. The large AraBERTv2, AraBERTv0.2, and ArabicBERT models performed better than their base versions at detecting inaccurate health tweets, as shown in Table 4. In contrast, the BERT-base models might be better at detecting accurate tweets, except for the AraBERTv2, whose large and base versions performed similarly.

Of the pretrained word embeddings, the results in Table 4 show that Mazajak Skip-Gram is the best based on *model accuracy* and F_1_ score.

**Table 4:** Comparison of the performance of machine learning models for detecting the accuracy of health-related tweets.

Model and class			Precision		Recall		F_1_ score		Macroaverage		Model accuracy
**AraBERTv2^a^-base**											
	Inaccurate	0.804		0.7627		0.7826		0.8279		0.8397	
	Accurate	0.86		0.8866		0.8731		0.8279		0.8397	
**AraBERTv2-large**											
	Inaccurate	0.8276		0.8136^b^		0.8205^b^		0.8564^b^		0.8654^b^	
	Accurate	0.8878		0.8969		0.8923		0.8564^b^		0.8654^b^	
**AraBERTv0.2^c^-base**											
	Inaccurate	0.8519		0.7797		0.8142		0.8543^b^		0.8654^b^	
	Accurate	0.8725		0.9175		0.8945		0.8543^b^		0.8654^b^	
**AraBERTv0.2-large**											
	Inaccurate	0.8448		0.8305^d^		0.8376^d^		0.8701^d^		0.8782^d^	
	Accurate	0.898^d^		0.9072		0.9025^d^		0.8701^d^		0.8782^d^	
**MARBERT**											
	Inaccurate	0.7759		0.7627		0.7692		0.8154		0.8269	
	Accurate	0.8571		0.866		0.8615		0.8154		0.8269	
**ARBERT**											
	Inaccurate	0.7903		0.8305^d^		0.8099		0.8447		0.8526	
	Accurate	0.8936		0.866		0.8796		0.8447		0.8526	
**QARiB**											
	Inaccurate	0.7797		0.7797		0.7797		0.8228		0.8333	
	Accurate	0.866		0.866		0.866		0.8228		0.8333	
**ArabicBERT^e^-large**											
	Inaccurate	0.8654		0.7627		0.8108		0.8532		0.8654^b^	
	Accurate	0.8654		0.9278^b^		0.8955^b^		0.8532		0.8654^b^	
**ArabicBERT-base**											
	Inaccurate	0.8913^d^		0.6949		0.781		0.83492		0.8525	
	Accurate	0.8364		0.9485^d^		0.8889		0.83492		0.8525	
**BLSTM^f^ Mazajak CBOW^g^**											
	Inaccurate	0.7719		0.7458		0.7586		0.8079		0.8205	
	Accurate	0.8485		0.866		0.8571		0.8079		0.8205	
**BLSTM Mazajak Skip-Gram**											
	Inaccurate	0.8542		0.6949		0.7664		0.8222		0.8397	
	Accurate	0.8333		0.9278^b^		0.8780		0.8222		0.8397	
**BLSTM ArWordVec Skip-Gram**											
	Inaccurate	0.8261		0.6441		0.7238		0.7919		0.8141	
	Accurate	0.8091		0.9175		0.8148		0.7919		0.8141	
**BLSTM ArWordVec CBOW**											
	Inaccurate	0.7925		0.7119		0.75		0.805		0.8205	
	Accurate	0.835		0.8866		0.86		0.805		0.8205	
**BLSTM AraVec CBOW**											
	Inaccurate	0.6865		0.7797		0.7302		0.7737		0.7821	
	Accurate	0.8571		0.866		0.8172		0.7737		0.7821	
**BLSTM AraVec Skip-Gram**											
	Inaccurate	0.7313		0.8305^d^		0.7777		0.8136		0.8205	
	Accurate	0.8144		0.8144		0.8494		0.8136		0.8205	
**BLSTM fastText**											
	Inaccurate	0.8158		0.5254		0.6392		0.7382		0.7756	
	Accurate	0.7627		0.9278^b^		0.8372		0.7382		0.7756	

^a^AraBERTv2: Transformer-based Model for Arabic Language Understanding version 2.

^b^Represents the second-best value.

^c^AraBERTv0.2: Transformer-based Model for Arabic Language Understanding version 0.2.

^d^Indicates the best value.

^e^BERT: bidirectional encoder representations from transformers.

^f^BLSTM: bidirectional long short-term memory.

^g^CBOW: Continuous Bag of Words.

## Discussion

### Principal Findings

As noted earlier, the examples given in the *Results* section showed that accurate tweets were more focused on preventive medicine, whereas inaccurate tweets were more focused on alternative and natural medicine. However, it could be argued that this is because of the keywords used in extracting and filtering the tweets or because of the selected tweet examples. Nevertheless, a previous study mentioned that the prevalence of natural alternatives and alternative medicine compared with medicine provided by the health care system [62] may be harmful. To illustrate the importance of this with respect to specific patients, there was a reported case of a patient with cancer who took alternative medicine promoted on SM, which caused the hospital to temporarily stop her cancer treatment to repair the damage caused by that medicine [63]. At a more general level, going forward, insights such as these could provide additional levers with which to detect inaccurate health tweets.

The results of BLSTM with pretrained word embedding models (AraVec, Skip-Gram, and Mazajak) are comparable with the results of some BERT models, including MARBERT, QARiB, and ArabicBERT-large. Indeed, this has been previously reported in the literature, where MARBERT and QARiB outperformed some of the other transformer models, such as ArabicBERT and AraBERT [55,56]. Again, a takeaway from this is that pretrained word embeddings might outperform pretrained BERT models in this first comparative study directed at Arabic. There is no guaranteed best model between pretrained word embeddings and pretrained transformer models for this language.

However, in general, the results showed the superiority of the BERT models over BLSTM with pretrained word embedding models. Overall, 19 best or second-best results were obtained by the 9 BERT-based approaches, whereas only 3 best or second-best results were obtained by the 7 pretrained word embedding models.

Most models performed better at detecting accurate health tweets than inaccurate tweets. The detection rate (recall) for accurate tweets ranged from 0.9485 to 0.8144. This means that most of the models missed only approximately 5% to 19% of the accurate tweets, which is a promising result. In contrast, the detection rate for inaccurate tweets was lower and had a wider range, from 0.8305 to 0.5254, implying that the best models missed up to 17% of inaccurate tweets. This is concerning as we would like to successfully identify all inaccurate tweets, and even the best model missed 17% of them.

The flip side of this is precision: how many accurate or inaccurate tweets identified by the technique are actually accurate or inaccurate. In terms of inaccurate tweets, the approaches ranged from 0.7759 to 0.89130—quite a large span, which means that if the wrong technique is chosen, approximately one-quarter of the tweets identified as inaccurate is incorrectly classified. Probably, more of a concern is the number of tweets identified as accurate that are not. Similarly, here, the span ranged from 0.8913 to 0.7627, again implying that if the wrong technique is chosen, this could be problematic.

Some models that had high detection rates for accurate health tweets could have low detection rates for inaccurate tweets. For example, the ArabicBERT-base and BLSTM fastText models were the best and second best for accurately detecting tweets, with success rates of 0.9485 and 0.9278, respectively. However, in detecting inaccurate tweets, BLSTM fastText had the lowest detection rate (52%) and the ArabicBERT-Base model had the second-lowest detection rate (69%). In other words, a practitioner who uses the best model for identifying accurate health tweets might miss approximately 30% to 48% of inaccurate tweets.

Similarly, the ARBERT and AraVec Skip-Gram models performed similarly to the AraBERTv0.2-large model in terms of precision when detecting inaccurate health-related tweets; however, these 2 models did not perform as well on the other metrics. For example, the AraVec Skip-Gram model had the second-lowest rate of *model accuracy* in classifying accurate tweets as inaccurate. Although the ARBERT model performed well compared with the BLSTM models, with regard to classifying accurate tweets as inaccurate, it had the third-lowest rate of *model accuracy* among the 9 BERT models tested in this study. In other words, the ARBERT models incorrectly classified accurate tweets as inaccurate at a higher rate than the 6 other BERT models, as shown in Table 4.

Ideally, a technique would provide high precision in both identification and recall; however, this did occur in the data set for accurate or inaccurate tweets. AraBERTv0.2-large came closest in this regard with high-accuracy tweet precision and recall, best recall for inaccurate tweets, and suboptimal precision for inaccurate tweets. Similarly, AraBERTv2-large performed quite well across accurate tweets but did not perform quite well on inaccurate tweets.

However, these models (AraBERTv0.2-large and AraBERTv2-large) consume relatively more resources, being based on BERT-large. Among the base models, AraBERTv0.2-base has an F_1_ score of 0.8543, which is good, and also has a similar *model accuracy* to AraBERTv2-large. These models can be considered as an alternative if resources are an important consideration.

Regarding the performance of pretrained word embeddings, we found that Mazajak Skip-Gram was the best. We made the same observation in our previous work on the detection of health-related tweets [31].

Finally, with respect to the accuracy of the best model in our study (ie, AraBERTv0.2-large), our results are satisfactory when compared with the results of previous studies [8-10] that make use of expert opinion. The F_1_ score of our best model was 87%, whereas the best F_1_ score reported in the study by Zhao et al [10] was 84%, as shown in Table 2. Furthermore, although these previous studies targeted a specific health topic (such as cancer [8] or autism [10]), we used a data set of tweets on a wide range of health care topics, suggesting that it would be more difficult to classify our data set.

It should be noted that all 3 studies with *model accuracy* or F_1_ scores >90% did not rely on expert opinion (Tables 1 and 2). In addition, 2 of these 3 studies [18,19] targeted a specific outbreak condition (COVID-19), and their models were trained on a larger data set (eg, Al-Rakhami and Al-Amri [18] trained their model on 409,484 tweets). For the third study [21], the keywords used to extract initial tweets were derived from 6 preidentified rumors related to Zika. The size and nature of the data used to train these models might explain why they seemed to achieve better accuracy than the model proposed here. In this study, we trained a model to detect the accuracy of generic health-related information, making the approach applicable to tweets that are more or less categorical in their labeling (as illustrated in the samples in Textbox 2).

### Limitations

This study only considered tweets agreed upon by experts. Although this helps us reduce the uncertainty in our data set, it might be a limitation as the model is not trained or tested on tweets that are more marginal—tweets about which the experts are unsure.

One of the strengths of this model is that it was trained on general health-related tweets. The accuracy of the model for each health condition or topic may vary, and future studies should evaluate the model for specific health topics.

All models used here are language dependent and might not be directly applicable to other languages. However, there are BERT alternatives for many languages, and there is evidence that BERT outperforms word embedding-based models. Therefore, we believe that this model could perform similarly in other languages.

Regarding the metrics used to evaluate the models, it should be noted that the F_1_ measure has been subjected to some criticism. Although we showed the F_1_ score for both classes (accurate and inaccurate health tweets), it should be noted that the measure gives equal importance to both classes (accurate and inaccurate health tweets). Moreover, the F_1_ score generally does not consider true negatives in its equation [64,65].

### Conclusions

The goal of this study was to develop and evaluate a state-of-the-art ML model for detecting the medical trustworthiness of health-related tweets in Arabic. To achieve this, we first constructed a labeled data set to train the classifiers. We then compared 2 different DL approaches for training a classification model, namely, 6 pretrained word embedding models as an input model for BLSTM and 11 pretrained transformer language models. The percentage of inaccurate health tweets in the data is approximately 38% (296/799), which is comparable with previous studies that used data sets with a number of inaccurate health-related tweets in the range of 30% to 50%. Our AraBERTv0.2-large model achieved 87.7% *model accuracy* on the test data set, which is satisfactory. Overall, our results clearly indicate that the AraBERTv0.2-large model outperforms the other models in detecting the medical accuracy of health-related tweets.

This study established an ML model to identify the accuracy of health-related tweets in response to the proliferation of health misinformation on SM. Although misinformation detection has been researched, only 1 study was concerned with detecting the accuracy of Arabic health-related tweets, and it was only for a specific topic (cancer). Furthermore, no DL model has been evaluated in prior studies to detect the accuracy of Arabic health-related tweets. In this study, we used a more extensive data set to develop a more general model using state-of-the-art ML models that have not been implemented before for this type of problem.

The potential of such work cannot be overstated. If a robust model can be built, it will allow for the detection and dissemination of accurate tweets. Similarly, this would allow for the flagging of inaccurate tweets. Both measures would significantly improve health information dissemination on Twitter. However, it should be noted that although this work will improve the situation, it will still inaccurately classify 13% of the tweets.

Moreover, the examples in Textbox 2 imply disparities between accurate and inaccurate information in terms of the topics covered across the data set—a trend supported by the informal sampling of that data set. Accurate tweets seem to be more preventive, whereas inaccurate health tweets seem to promote *natural* and alternative medicine. Thus, it might be more feasible to develop a model for detecting health topics in combination with a model for detecting the accuracy of health information and thus improving accuracy.

To further improve the accuracy of the developed model, ensemble learning can yield better results by combining models that perform well (ArabicBERT-large, ARBERT, and AraVec Skip-Gram). However, ArabicBERT and AraBERTv0.2 were trained on a similar corpus, as shown in Table 3. Another approach could be to combine models pretrained on different corpora, such as ArabicBERT-large and MARABER (ArabicBERT pretrained on Wikipedia articles and news articles; MARBERT pretrained on 1 billion tweets).

## Appendix

Multimedia Appendix 1 Tweet IDs with their labels used in the study.

## Abbreviations

AraBERT: Transformer-based Model for Arabic Language Understanding
AraBERTv0.2: Transformer-based Model for Arabic Language Understanding version 0.2
AraBERTv2: Transformer-based Model for Arabic Language Understanding version 2
BERT: bidirectional encoder representations from transformers
BLSTM: bidirectional long short-term memory
DL: deep learning
LSTM: long short-term memory
ML: machine learning
NLP: natural language processing
RF: random forest
SM: social media
SVM: support vector machine
WHO: World Health Organization
